# Design of Repository and Search Platform for Art Painting Teaching Resources in Universities Based on Model of Decision Tree

**DOI:** 10.1155/2022/1366418

**Published:** 2022-08-25

**Authors:** Yuling Liu

**Affiliations:** Solux College of Architecture and Design, University of South China, Hengyang 421001, Hunan, China

## Abstract

At present, art education curriculum reform is in full swing. Art education in China started late but as an important element of quality education. Art education is particularly important today with rapid economic development and advanced technology. The art subject is the sum of the teaching content of the art subject and the execution of teaching activities expressed through the network, including two components: the teaching content organized according to specific teaching objectives and teaching strategies and the network teaching support environment. Unlike existing multimedia courseware, several features reflect the advantages of online education, unlike online courseware. Each school generates a large amount of student performance data every year, and existing systems only perform simple backups, queries, and statistics on these data, which do not reflect the weaknesses of education in a centralized and comprehensive manner. Data mining can extract valuable information hidden behind the data from a large amount of data and has been applied in more and more fields with good results, which helps people to make correct decisions. In this study, we propose a decision tree model-based repository and search platform for school art and painting education resources, briefly outline the decision tree model, and analyze the architecture and operation of the school art and painting education resources repository and search platform. The experimental results show that the Virginica accuracy of the decision tree model is 0.42 higher than that of C4.5, which greatly improves the accuracy of the preferred class and ensures the overall accuracy remains unchanged. Therefore, this system realizes the art education resource repository system and realizes the needs of college users to share art professional information and resources.

## 1. Introduction

The rapid development of China's market economy and so the demand for art are growing [1]. Combining the current situation of art education in China with the real problems of art education curriculum reform and proposing new ideas, the field faces continuous, systematic, and in-depth research [2]. The School of Fine Arts drawing resource repository is an important platform to meet the demand for art resources of art majors in schools, and schools can use this platform to play the role of art resource sharing and bring together various resources of art drawing [3]. Since the school drawing resource storage requires high repository and search efficiency, while the picture resource files are large (stored in RDF ternary format) and many process files, system scalability is one of the most noteworthy indicators for this resource [4]. The Internet has a wealth of multimedia information resources, including text, images, sound, video, and other multimedia information resources [5]. However, it also causes problems such as the scattered distribution of resources and disorganization [6].

The ever-increasing amount of information places higher demands on data storage, management, and analysis [7]. This requires new tools that can automatically transform data and convert it into valuable information and knowledge [8]. Starting from the storage capacity problem, information silos and hotspots emerge. This is because information silos and hotspots are independent individuals that do not share information effectively [9]. Therefore, it is necessary to integrate and manage media resources in schools to provide rich teaching and learning resources for teachers and students to improve teaching and research in high schools and further improve the quality and effectiveness of teaching and learning [10]. However, among the many resource sites in a school or district, system integration differs significantly in the non-standardization of information transfer protocols and information formats, the establishment of each resource repository on the system platform, database selection, and the way resources are classified. Although regional networks share educational resources, they are unable to share and access resources between different platforms and systems [11].

Data access increases because clients have to specialize in parsing different data structures when accessing data [12]. Concurrent connection time and resource display speed are greatly affected [13]. Decision trees are an important classification and prediction method that can handle both discrete and continuous data and are easy to understand [14]. There have been significant changes in the nature of information retrieval, which can be reflected in more diverse, open, dynamic and widespread deployments, faster update rates, faster change rates, network delivery processes, and network management [15]. However, as the number of components in the component repository increases, users may experience difficulties in finding and selecting components, thus requiring an efficient organization and management of the component repository, which is directly related to the success of reuse cases.

The innovations of this study are as follows:

1. This study introduces the teaching resources of school art education as an aspect of the art education support system in the whole system and provides a macro and holistic analysis within the framework of the art education support system.
2. The system design of this study is based on the design requirements of software engineering and uses advanced technology to achieve effective sharing of the networked multimedia network.
3. Extensive real-world data collection combined with the model of decision tree depicts a more comprehensive and practical development of Chinese school art education and educational resources.

The research framework of this study consists of five parts, which are structured in detail as follows.

The first part of the study introduces the background and significance of the research and describes the main tasks of the study. The second part introduces the work related to the resource repository and search platform and decision tree model of the Department of Fine Arts and Painting. The third part classifies the system operation architecture design and system software architecture so that readers of this study can have a more comprehensive understanding of the resource repository and search platform for picture professors based on the decision tree model. The fourth part is the core of the study, from the analysis of the construction of the decision tree in the resource repository and search platform, and the analysis of the splitting attribute selection of the decision tree in the retrieval process, to complete the description of the application analysis of the model of decision tree in the design of the teaching resource repository and search platform. The final part of the study is a summary of the full work.

## 2. Related Work

### 2.1. Fine Art Painting Teaching Resources Repository and Search Platform

The rapid development of China's economy, the significant increase in comprehensive national power, the improvement of people's living standards, and the enrichment of material life inevitably bring a high level of demand for spiritual life, in which more and more people are involved. Art is gradually descending from the top of the pyramid and is no longer the behavior of a few “elites,” but is becoming more and more popular. Architectural art education resource storage effectively manages art-related basic data resources and multimedia information resources and provides teacher resources data and platforms for school art professional education and distance art education.

Waldhor proposed a web-based educational technology standard with Chinese characteristics (CELTS). Specifications and standards were mainly developed to describe the attributes of resources, such as media data, courseware, literature data, test papers, question banks, cases, online courseware, and FAQs [16]. From the perspective of resource construction and management, Huizhen classified online teaching resources according to various manifestations. In his opinion, whatever is conducive to the transfer of teaching resources is used as the primary reference for classification and representation [17]. Zheng and Zhou researched and designed a web-based educational resource repository, the main idea of which is to build an educational resource repository through the joint participation of users. The repository provides users with high-quality resources and also encourages them to upload their own resources to build a diverse repository of users and achieve sustainable growth of resources [18]. Starting from real school education, Sharma et al. classified online educational resources according to specific areas of school education and learners of different grades. As resources are presented in different ways on the online platform, the subcategories of their associated metadata must also have different priorities [19]. Mimis et al. introduced Web service technology into the development process of educational resource sharing systems and designed corresponding intermediate application components to access heterogeneous teaching resource databases, thus solving the problem of accessing scattered and isolated heterogeneous resource databases on campus [20].

As China's education system continues to improve and the academic field continues to mature and grow, the long-established field of art is receiving more and more attention. Relevant policies and regulations have been introduced one after another, providing assurance that art education can continue to develop.

### 2.2. Model of Decision Tree

With the change of the times, Chinese art education has undergone significant changes at all levels, in all dimensions, and in all aspects. Some old educational concepts of the past need to be changed urgently, and some factors are constrained and affect healthy development. All decisions in human society are inclusive and complex, and in addition to cost factors, other influences and factors can be considered, such as profit-sensitive decision problems need to consider profit factors and preference-sensitive decisions. The causes of the problem need to be considered and taken into account.

Yong and Yan used a decision tree-based classification mining method to analyze the data in the student achievement database and applied the algorithm in the analysis of student achievement to build a decision tree model of professional competence so that teachers and school education decisions conform to those produced by the creators and can gain insight into the problems in education, so they can use the information provided by grades to optimize education and educational planning and decision-making [21]. In order to obtain an ideal decision tree model, Niu et al. conducted an in-depth study of the attribute selection and pruning optimization in the construction process, such as the impact of the decision tree cost model on the actual decision-making process [22]. Lu and Zhou propose the idea of combining data mining techniques with statistical analysis for multi-strategy design. We used a decision tree-based classification mining approach to analyze data in a student grade database to create intuitively displayable decision trees of student grades. In other grade calculation methods, the location of specific grades provides assessment information for the teacher department [23]. Xie et al. used the principles of determining cost-sensitive classification learning and balancing costs to create an objective function with the goal of minimizing costs [24]. Cai and Wu introduced the classical association rule Apriori algorithm and the well-known decision tree algorithm ID3 and used the association rule algorithm to find out the effect of excellence in one course on another course [25].

The decision tree model can obtain various knowledge needed to support decision-making from a large amount of data in the component repository and uses data mining techniques to construct and reuse mining datasets based on historical information and feedback from component reuse. It uses this data mining method to mine some component reuse rules and exclude human factors as much as possible.

## 3. The Repository and Search Platform of Painting Teaching Resources Based on Model of Decision Tree

### 3.1. System Operation Architecture Design

**Figure 1:**
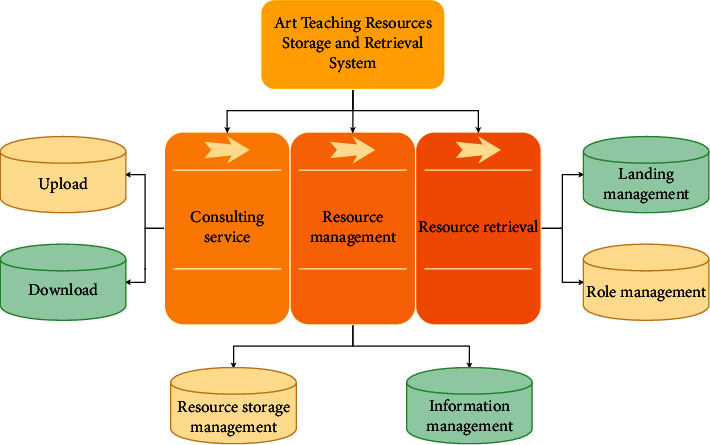
Functional requirements of teaching resource repository platform.

There are still many problems in conducting research on the repository and search platform for art painting resources in schools, which makes it difficult for them to bring out their unique advantages under the influence of the traditional model [26]. In traditional art class teaching, students' learning resources mainly consist of learning materials and textbooks prepared by teachers, which are slightly insufficient compared with learning resources on the Internet [27]. Moreover, since most of these materials end with the completion of the teacher's teaching time, students are unable to reorganize their knowledge and rely mainly on the image storage in their minds when completing assignments [28]. In the material management and sharing system, the entities oriented are mainly users and resources. Accordingly, the functional requirements of the system are divided into two modules: user management and material resource storage. The functional requirements of the teaching resource repository platform are shown in Figure 1.

The first is the backend program, which handles the business operations and data processing of the system and realizes the entry of material resource data into the database. The decision tree is constructed by growing the tree several times, specifically by dividing the training samples in a dataset to form a tree structure. It only constructs a decision path to classify each test case:

(1) $$\begin{array}{c} \text{Average Gain}\left(A,T\right)=\frac{\text{Gain}\left(A,T\right)}{\left|A\right|}, \end{array}$$

*A* is the attribute, |*A*| is the number of nodes, and Gain(*A*, *T*) is the reduced misclassification cost.

There are three subcomponents for data generation and pre-processing, the data generator creates data in RDF/XML serialized format and uses the *N* Triple Converter component to fetch this data and convert it to *N*-Triples serialized format. And the fuzzy split entropy minimum condition property is selected as an extension property. Where:

(2) $$\begin{array}{c} FE\left(D,A_{i}\right)=-\sum_{j=1}^{k_{i}}\frac{m_{i_{j}}}{m_{i}}E\left(D\cap A_{ij}\right), \end{array}$$

*D* is the leaf node, *A*_*i*_ is the unused conditional attributes, and *FE*(*D*, *A*_*i*_) is the fuzzy partition entropy.

Art education in basic education still belongs to the category of “quality education” [29]. However, art education at higher levels has some characteristics of its own, and the subject matter has increased, and the requirements for teaching resources are relatively high [30]. Therefore, professional art resource teaching websites can provide a relatively wide range of knowledge, and most of them are carefully organized by teachers and provide links to other related online learning resources. Students can find suitable learning resources more easily, which greatly enrich the content of art teaching and provide a new way to cultivate students' creative ability.

Next is the user interaction interface, where users submit business processing requests to the system within their rights, and the control engine controls the order of business processing and coordinates the invocation of functional functions. The component uses the Jena framework to convert data, and a predicate-based file splitter gets the converted data and splits it into predicate files. The splitting attribute selection factor for defining the node attributes is then as follows:

(3) $$\begin{array}{c} \text{ASF}\left(A\right)=\left(2^{\text{Average Gain}\left(A\right)}-1\right)*\text{Incr}-EP\left(A\right). \end{array}$$

Average Gain is the average information benefit and Incr − *EP*(*A*) is the increment of attribute.

This kind of educational material resources needs to be uploaded in a package in advance before they can be downloaded, and the relevant properties need to be described when submitting the resources. Then even in the case of incomplete training examples, it is possible to learn useful hypotheses from the set of training examples when large to enable the correct classification of unknown examples. The EP increase needs to be defined before giving the formula for the split attribute selection factor:

(4) $$\begin{array}{c} \text{Incr}-EP\left(A\right)=\sum_{i=1}^{n}EP\left(A_{i}\right)-EP, \end{array}$$

∑_*i*=0_^*n*^*EP*(*A*_*i*_) is the sum of effective preferences of all child nodes split by splitting attribute *A* and *EP* is the effective preference of nodes without attribute *A* as split attribute.

**Figure 2:**
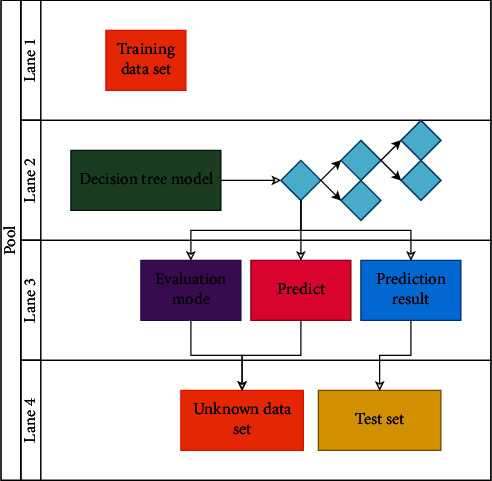
Model of decision tree.

Any non-leaf node of the decision tree represents a partition of the dataset on an attribute, and the dataset corresponding to the corresponding node is split into subsets by the difference in attribute values, and each subset is represented by a branch. The model of the decision tree is shown in Figure 2 below.

When examining the support system of art education, we need to dynamically link and integrate the originally static and independent factors, so that we can grasp the impact of the various “combined forces” on art education from a macro perspective and systematically analyze each link.

Finally, there is a database in which material resource information, user information, etc. are stored. The predicate-based files are then fed into an object type-based file splitter which splits the predicate files into smaller files based on the object type, and then the output of the last component is put into HDFS. the amount of information required for a decision tree to make a correct category judgment, for example, is:

(5) $$\begin{array}{c} I\left(p,n\right)=-\frac{p}{p+n}{\text{log}}_{2}\frac{p}{p+n}-\frac{n}{p+n}{\text{log}}_{2}\frac{p}{p+n}, \end{array}$$

*p*, *n* is the size of positive and negative examples in vector space.

Each split from the root node to the leaf node is unfolded according to some attribute, and each non-leaf node chooses an attribute in the decision process and generates different branches according to different attributes. If a user module adopts the schema, teaching materials are submitted, the directory structure where they are located should be noted, and for the schema, teaching materials submitted online, the same changes in the relative directory structure should be noted. The test attributes of the inner nodes of a decision tree may be univariate, i.e., each inner node contains only one attribute, or multivariate, i.e., there are inner nodes that contain multiple attributes.

### 3.2. System Software Architecture

**Figure 3:**
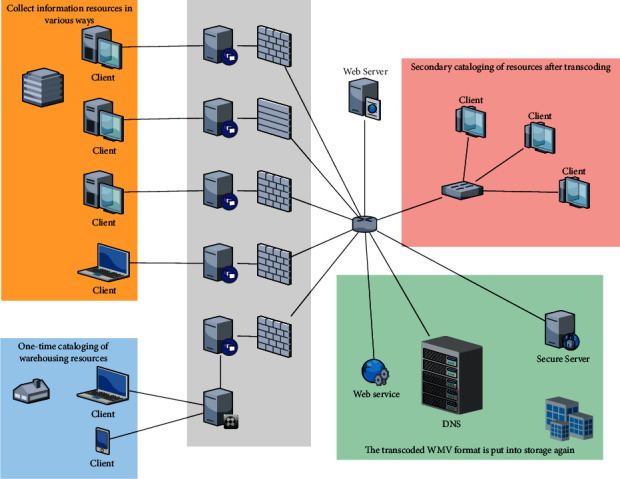
System architecture diagram of front-end resource warehousing and resource storage.

In the whole architecture of the model of decision tree-based painting teaching resource repository and search platform, the control engine is the core of the whole software architecture with other parts interconnected to coordinate and control data processing and business operations and realize communication with users. The system architecture of the front-end resource entry and resource storage part is shown in Figure 3.

The first step is to start the central controller to detect the BUF size of the structure and communication transfer to ensure that the data transfer will not lead to communication errors due to data overflow and other problems. In the model of decision tree, we redefine the split attribute selection factor:

(6) $$\begin{array}{c} ASF\left(A_{i}\right)=\frac{\left(2^{\text{Average gain}\left(A_{i}\right)}-1\right)}{TC{\left(A_{i}\right)}_{\text{normal}}}*\text{Incr}-\text{UCB}\left(A_{i}\right), \end{array}$$

*A*_*i*_ is the *i* attribute in the set *A*, Average gain(*A*_*i*_) is the average information gain, *TC*(*A*_*i*_)_normal_ is the standardized test cost, and Incr − UCB(*A*_*i*_) is the UCB dosage.

In HDFS, files take up space replication factor size. Since RDF is text data, HDFS needs a lot of space to store the files. The interaction between multiple tasks, which are various “request-response” relationships, is achieved by the client sending a data processing request to the server, requesting the server to complete it, and returning the result to the client. The content of art teaching should reflect the spirit of the times and adapt to the trend of social development. Make full use of the local art resources to enrich the content of art teaching. The content of computer art and pottery can be added in places with conditions. So from the hand of art painting management, we set up electronic files of painting data metadata and digital documents by designing data management system, so as to facilitate the accession and management of digital painting data. It is assumed that the distribution of index words in unrelated documents can be approximated by the distribution of index words in all documents in the information set. That is,

(7) $$\begin{array}{c} \left\{\begin{array}{c} P\left(t_{i}|R\right)=0.5,\\ P\left(t_{i}|\bar{R}\right)=\frac{n_{i}}{N}, \end{array}\right. \end{array}$$

*n*_*i*_ is the number of documents containing index words and *N* is the total number of documents in information set.

Next is the front-end request processing, the administrator sends the system login request by user name and password, the central controller verifies the user name and password, returns the authentication result, and assigns the corresponding permission according to the user role. Suppose a decision tree is constructed for a data set with two class attribute values, and there are *p* positive samples and *n* negative samples at a node, then the class labeling method for this node is as follows:

(8) $$\begin{array}{c} \left\{\begin{array}{c} P,\text{ifp}\times FN>n\times FP,\\ N,\text{ifp}\times FN<n\times FP, \end{array}\right. \end{array}$$

*FN* is the price to be paid when the positive node is wrongly judged as the negative example, *P* is the node judged as a positive node, *FP* is the price to be paid when the counter-example node is wrongly judged as a positive example, and *N* is the node judged as a counter-example node.

To minimize the amount of space, the public prefix in the URI is replaced with some much smaller prefix string and this prefix string is tracked in a separate prefix file. This greatly reduces the amount of space required for the data, and since there is no cache in Hadoop, each SPARQL query needs to read the file from HDFS. Entropy is a concept of measuring the amount of information, in information theory, it represents the uncertainty of random variables and it focuses on solving the problem of quantifying information. The formula is as follows:

(9) $$\begin{array}{c} P\left(X=x_{i}\right)=p_{i},i=1,2,\ldots ,n, \end{array}$$

*X* is the discrete random variable and *P*(*X*=*x*_*i*_) is the probability distribution of discrete random variables.

The school library should be equipped with art books and other art resources, including teachers' reference books, students' reference books, art magazines, art education magazines, slides, CD-ROMs, etc., for teachers to prepare and teach classes, and for students to collect and consult materials and for self-learning or cooperative learning. Out of consideration for the huge amount of school art painting materials, this system will adopt the full-text image retrieval method to store and retrieve digital images of school art paintings.

Finally, after adding, deleting, or updating the resources, the material shared directory will be changed to some extent, and the changed shared directory will be sent to the central server and other node servers for updating the shared directory. The reason for not storing data in a single file is that in Hadoop files are the minimum input unit for MapReduce jobs. If all the data is placed in a single file, then the entire file will be input to the MapReduce job for each query. Model of decision tree always selects the attribute with the largest information gain among all attributes as the split attribute for the current node. The formula to calculate the classification of a given sample is as follows:

(10) $$\begin{array}{c} I\left(s_{1},s_{2},\ldots ,s_{m}\right)=-\sum_{i=1}^{m}p_{i}{\text{log}}_{2}\left(p_{i}\right), \end{array}$$

*S* is the collection of sample data, *m* is the number of distinct values, *C*_*i*_ is the category, *S*_*i*_ is the sample number, and *p*_*i*_ is the probability that any sample belongs to *C*_*i*_.

Database design is divided into physical database design and logical database design. Logical database design is to design the global logical structure of the database according to the demand of system construction, so as to respond to the business logic. The physical database design is to design and implement the storage structure and access method of the database according to the logical structure of the database. The full-text image search method is used to realize the search of a large number of images of school art and painting resources, so as to improve the efficiency of the system in retrieving images. The RDF type files were also divided into as many files as the number of different objects that the RDF: Type predicate has. This further reduces the amount of space required to store the data.

## 4. The Application Analysis of Model Decision Tree in the Design of Teaching Resources Repository and Search Platform

### 4.1. Construction and Analysis of Decision Tree in Resource Repository and Search Platform

**Figure 4:**
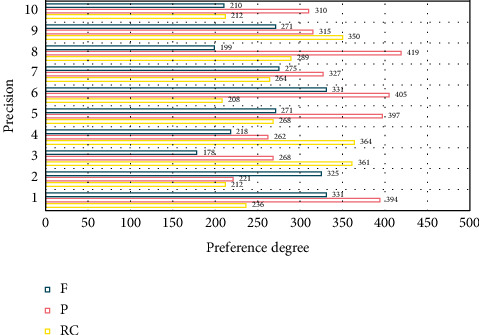
The influence of preference degree on the accuracy of decision tree with preference cost.

Decision tree-based learning algorithms have the advantages of being fast and accurate, generating understandable rules, being relatively inexpensive, handling continuous and discrete-valued attributes, and showing clearly which attributes are important. In addition, the user does not need to know much background knowledge during the learning process, and the algorithm can be used to learn as long as the training examples can be expressed in an attribute-to-conclusion style. To construct a decision tree, a training sample set with class labels is provided, and then the training sample data space is partitioned by a decision tree classification algorithm, resulting in an inverted tree structure. The cost of predicting versicolo as a preference class is 5, and the cost of predicting setosa as a preference class is 400, the preference cost of the latter is much larger than the former, and the main juggling of the sent sample setting is to tell the algorithm that predicting setosa as a preference class is intolerable behavior. The experimental results are shown in Figure 4.

**Figure 5:**
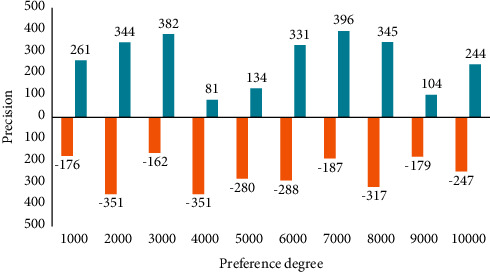
Comparison of *F* value before and after adding preference cost.

First, the tree is generated, starting with all data at the root node, and then the data is recursively sliced. The problem of understanding software reuse is a key issue that affects the success of reuse. In other words, in the actual soft component reuse process, the reusers should not only be able to retrieve the relevant set of components efficiently but also the key is how to make the reusers understand the retrieved components quickly so as to find their real needs in this set of components, which is a complex decision process. Calling the *F*-value before joining the preference cost as *F*1 and the *F*-value after joining as *F*2, the comparison of *F*1 and *F*2 is shown in Figure 5.

Data noise elimination, deceptive data, etc., all of which affect the accuracy and practicality of the final generated decision tree and rule extraction, i.e., its application scope is somewhat limited. So the training sample data can reside on disk and only a part of it is loaded into memory. The process of server retrieval is not a process of division of labor, independent search, and processing of one-sided information, and the retrieved information is returned to the server that sent the retrieval request, aggregated, and organized to achieve macro synchronous retrieval.

Next is the tree pruning, which is to remove some data that may be noisy or abnormal. Then the initial training data set is divided into several disjoint subsets according to the selected splitting points or splitting subsets, and these subsets are used to select the training set with splitting attributes by the branching nodes formed after the splitting of the root node. And the generated branching points are split by the same method until the resulting child nodes of the split are all leaf nodes labeled with classes. The test cost for each attribute was randomly assigned between [20, 90], and the cost and gain matrices for each data set were set by reference to manual settings. The test results based on the information gain rate criterion are shown in Table 1.

The samples in the test set are input to the generated decision tree one by one, and then the predicted class number of each sample is compared with the actual class number, and the results of the comparison are counted to obtain the accuracy of the decision tree classification. Users may not have a clear goal before querying, but just want to search the soft component library to see if there are components that can be utilized and reused, so it is necessary to provide users with a certain degree of help through the reuse history of components and the reuse experience of other users.

Finally, the condition that the decision tree stops partitioning has a node on which the data all belong to the same class no more attributes can be used to partition the data. That is, a certain attribute is selected at a node to divide the construction of different branches according to some rule. Component feedback information can enable the component reusers to get some reuse experience information from previous component reusers, enhance the understanding of the components and reduce the reuse workload. Efforts should be made to promote the integration of information technology with the teaching of other subjects, to encourage the application of information technology tools in the teaching of other subjects, and to integrate information technology education into the learning of other subjects. Therefore, the information gain is used as the criterion for attribute selection, so that the largest category information of the tested examples can be obtained when each non-leaf node is tested, and the moisture value of the system is minimized after using this attribute to divide the example set into subsets.

### 4.2. Analysis of Split Attribute Selection of Decision Tree in Retrieval Process

Educational resources are constantly being added, which makes it difficult to retrieve them. At present, information retrieval technology has been developed to a very mature stage, and has reached the network and intelligence.

**Figure 6:**
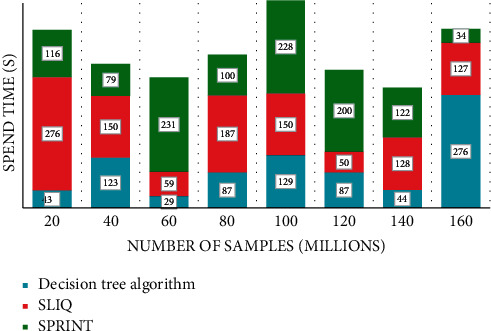
Comparison of running time of decision tree algorithm.

First of all, we use average gain instead of traditional information gain to enhance the classification ability of information gain. Information gain or information gain ratio is an important reference data for selecting feature values, and the data classification of the decision tree is to order a large amount of disordered data. The GINI index is used as the criterion for judging the merit of segmentation, and the computation of the GINI index is much smaller than that of the information gain ratio. The decision tree algorithm is compared with the SLIQ algorithm and SPRINT algorithm in a stand-alone environment, i.e., in a serial algorithm. The results are shown in Figure 6.

Search all the internal and external data information related to the business object and select from it the data that is suitable for data mining applications. If the correct rate obtained by classifying the sample data with the features we select is about the same as the correct rate obtained by random classification, then the value of this selected feature is indistinguishable, and not using such a feature for classification does not have the slightest effect on the accuracy of decision tree classification. This metric is called the attribute selection metric or the split merit metric. The attribute with the highest information gain (or maximum decompression) is selected as the test attribute for the current node, which minimizes the amount of information required to classify the samples in the resulting split and reflects the minimum randomness or “impurity” of the split.

**Figure 7:**
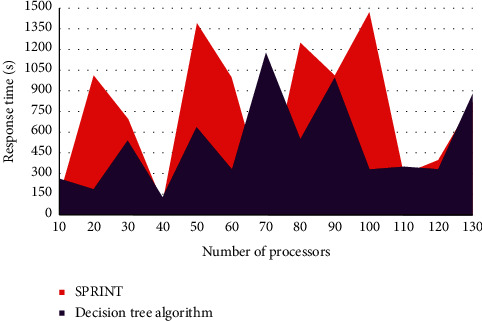
Comparison with SPRINT in parallel environment.

Second, the degree of the decision maker's preference for the class is considered in the selection of the splitting attribute, and an assumption exists on the selection of the maximized ASF attribute as the splitting attribute using mutual information as the feature selection amount. That is, the proportion of positive and negative examples in the training example set should be the same as the proportion of positive and negative examples in the actual problem domain. However, in general, the same cannot be guaranteed, so that there is a bias in computing the mutual information of the training set. Thus the optimization of the retrieval method in the real sense can be accomplished only on the basis of a reasonable representation of the resources. To check the performance of this design in a parallel environment, we compare the design of this study with the SPRINT algorithm which has good parallel performance. The SPRINT in a parallel environment is shown in Figure 7.

Convert the data into an analytical model. This analytical model is built for the mining algorithm. Building an analytical model that really fits the mining algorithm is the key to successful data mining. But instead of using information gain to measure the best split, it uses the X-test used in the column table to determine which category prediction attribute is the most independent from the predicted value. The model of the decision tree is compared with the decision tree built by the C4.5 algorithm on Iris, and the comparison results are shown in Table 2.

As can be seen from Table 2, the Virginica accuracy of the model of decision tree is improved by 0.42 compared to C4.5, which also significantly improves the accuracy of the preference class and ensures the same overall accuracy.

**Figure 8:**
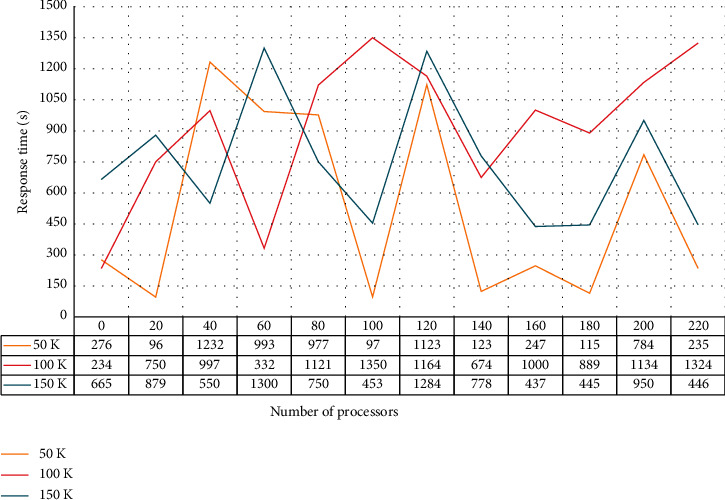
Scale up performance of decision tree.

Finally, the number of minimum support terms per branch of the decision tree needs to be predetermined before pruning, and then each subtree is detected from the top down. Then the information gain as the attribute selection benchmark is dropped and replaced by the information gain rate, which is more reasonable for attribute selection. Since the number of samples assigned to each machine is constant, the ideal goal is that the response time of the algorithm remains constant as the number of processors increases. The Scale up performance of the decision tree is shown in Figure 8 below.

That is, some nodes and subtrees are removed by pruning operations to avoid “overfitting” and thus eliminate anomalies and noise from the training set. So a path from the root to the leaf nodes corresponds to a merging rule, and the whole decision tree corresponds to a set of parsing expression rules. Thus, we can see that the information gain from feature improvement is the greatest, and the feature improvement can be selected as the root node or inner node in the feature selection of the decision tree. There were no remaining attributes that could be used to further divide the sample. In this case, majority voting is used. This involves converting a given node into a leaf and labeling it with the class in which the majority of the sample resides, or the class distribution in which the sample of nodes can be stored.

## 5. Conclusions

In recent years, China's art education reform has made remarkable achievements, and most domestic art educators have invested a lot of energy in both theory and practice. By organizing existing teaching materials and building an art resource room, we can collect and manage a large number of excellent teaching materials, realize the sharing and management of materials, and provide richer and more practical materials for the majority of users. In addition, with the advent of the era of big data, the complexity of the data environment is rapidly increasing as the amount of data accumulated by people is rapidly increasing. Traditional processing methods are facing new challenges, using the rules and laws of decision tree models to provide meaningful information for educational management decisions and help educational administrators allocate educational resources. Coordinating educational programs, reforming teaching practices and curricula, and making relevant educational management decisions. Therefore, this study analyzed and discusses the design of a decision tree model-based repository and search platform for school art and painting materials, aiming to realize the integrated storage and management functions of multimedia data resources. The article proposes a repository and search platform for art painting images and enables the sharing of painting resources among different schools, allows fast retrieval of a large number of RDF files, and describes the algorithm with an example, which also provides a reference for the current development of art painting in schools.

## Figures and Tables

**Table 1:** Test results based on information gain rate standard.

Data set	Iris	Wine	Glass
Misclassification cost	453	556	768
Profit	16577	21756	32445
UCB	34.25	25.16	19.67
Classification accuracy	97.23	67.45	32.19

**Table 2:** Comparison results.

	Decision tree model	C4.5
Virginica accuracy	0.98	0.56
Overall accuracy	0.93	0.67
Variance ratio	0.896	0.453

## Data Availability

The dataset is available upon request.

## References

[1] Ma G., Zhang L., Cui G., Cheng Y. (2019). Design of medical examination data mining system based on decision tree model. *Journal of Physics: Conference Series*.

[2] Nalinipriya G., Maheswari K. G., Kotteswari K. (2017). An enhanced priority scheduling algorithm for multi-server retrieval cloud system. *Journal of Information Science and Engineering*.

[3] Liu X., Zhu F., Fu Y., Liu Q. (2020). A resource retrieval method of multimedia recommendation system based on deep learning. *International Journal of Autonomous and Adaptive Communications Systems*.

[4] Kimi M. (2018). Storage and retrieval of multimedia resources in multimedia libraries: a study. *IASLIC Bulletin*.

[5] Chung E., Lee H. F. (2007). A generalised sequencing problem for unit-load automated storage and retrieval systems. *International Journal of Industrial and Systems Engineering*.

[6] Mao Z., Zheng T., Lian Z. (2021). Information system construction and research on preference of model by multi-class decision tree regression. *Journal of Physics: Conference Series*.

[7] Mokashi U. M., Suma V., Christa S. (2020). Regression and decision tree approaches in predicting the effort in resolving incidents. *International Journal of Business Information Systems*.

[8] Devasenapathy K., Duraisamy S. (2017). Evaluating the performance of teaching assistant using decision tree ID3 algorithm. *International Journal of Computer Application*.

[9] Tucker B. V., Kelley M. C., Redmon C. (2021). A place to share teaching resources: s. *Journal of the Acoustical Society of America*.

[10] Zav’yalova O. S. (2019). On the relationship between teaching objectives and teaching resources in the electronic course on extensive and intensive reading for foreign students. *Tomsk State Pedagogical University Bulletin*.

[11] Cao Z. (2020). Classification of digital teaching resources based on data mining. *Ingénierie des Systèmes d’Information*.

[12] Sun X. Z., Li S. Y., Tian X. Y., Hong Z., Li J. X. (2019). *Clinical Hemorheology and Microcirculation*.

[13] Du Y., Zhao T. (2021). Network teaching technology based on big data mining and information fusion. *Security and Communication Networks*.

[14] Behera H. S., Nayak J., Naik B., Ajith A. (2019). Advances in Intelligent Systems and Computing. *Computational intelligence in data mining Advances in Intelligent Systems and Computing*.

[15] Wei H. (2019). Research on the development of ideological and political education resources for college students based on the data mining technology. *International English education research: English version*.

[16] Waldhor K. (2019). Machine learning paradigms: advances in data analytics. *Computing reviews*.

[17] Huizhen J.  (2018). Evaluation of british and American literature teaching quality based on data mining. *IPPTA: Quarterly Journal of Indian Pulp and Paper Technical Association*.

[18] Zheng C., Zhou W. (2021). Research on information construction and management of education management based on data mining. *Journal of Physics: Conference Series*.

[19] Sharma S., Mahajan S., Rana V. (2019). A semantic framework for ecommerce search engine optimization. *International Journal of Information Technology*.

[20] Mimis M., Es-saady Y., El M., Ouled A. (2018). Adapted regulation level’s flipped classroom using educational data-mining. *International Journal of Computer Application*.

[21] Yong N., Yan Z. (2020). On-line classroom visual tracking and quality evaluation by an advanced feature mining technique. *Signal Processing: Image Communication*.

[22] Niu L., Chen X., Xu R. (2019). Quantitative analysis of the influence of learning resource scheduling in MOOC mode on traditional education and teaching. *International Journal of Continuing Engineering Education and Life Long Learning*.

[23] Lu L., Zhou J. (2021). Research on mining of applied mathematics educational resources based on edge computing and data stream classification. *Mobile Information Systems*.

[24] Xie Y., Wen P., Hou W., Yingdi L. (2021). A knowledge image construction method for effective information filtering and mining from education big data. *IEEE Access*.

[25] Cai W., Wu H. (2017). 33.Research on the optimal allocation of intellectual resources based on data mining analysis. *Boletin Tecnico/technical Bulletin*.

[26] Ai J., Gao J., Du P. (2019). Analyse the influence of big data on students’ learning behavior. *IOP Conference Series: Earth and Environmental Science*.

[27] Lino A., Rocha A., Macedo L., Sizo A. (2019). Application of clustering-based decision tree approach in SQL query error database. *Future Generation Computer Systems*.

[28] López M. B., Alor-Hernández G., Sánchez-Cervantes J. L., Salas-Zárate M. D. P. (2018). EduRP: an educational resources platform based on opinion mining and semantic web. *Journal of Universal Computer Science*.

[29] Rattanalerdnusorn E. (2018). Recbd approach - retrieval of efficient and content based relative data. *International Journal of Computational Intelligence Research*.

[30] Xiao Q. (2020). Resource classification and knowledge aggregation of library and information based on data mining. *Ingénierie des Systèmes d’Information*.

